# Dimensionality of the system usability scale among professionals using internet-based interventions for depression: a confirmatory factor analysis

**DOI:** 10.1186/s12888-020-02627-8

**Published:** 2020-05-12

**Authors:** Mayke Mol, Anneke van Schaik, Els Dozeman, Jeroen Ruwaard, Christiaan Vis, David D. Ebert, Anne Etzelmueller, Kim Mathiasen, Bárbara Moles, Teresa Mora, Claus D. Pedersen, Mette Maria Skjøth, Luisa Peleteiro Pensado, Jordi Piera-Jimenez, Didem Gokcay, Burçin Ünlü Ince, Alessio Russi, Ylenia Sacco, Enrico Zanalda, Ane Fullaondo Zabala, Heleen Riper, Jan H. Smit

**Affiliations:** 1 Department of Research and Innovation, GGZ inGeest, Specialized Mental Health Care, Oldenaller 1, 1081 Amsterdam, HJ Netherlands; 2 Department of Psychiatry, Amsterdam Public Health Research Institute, VU University Medical Center, Van der Boechorststraat 7, 1081 Amsterdam, BT Netherlands; 3 Department of Clinical, Neuro and Developmental Psychology, Clinical Psychology Section, Vrije Universiteit Amsterdam and the Amsterdam Public Health Research Institute, Van der Boechorststraat 1, 1081 Amsterdam, BT Netherlands; 4 Friedrich-Alexander-Universität Erlangen-Nürnberg, Schlossplatz 4, 91054 Erlangen, Germany; 5 Schön Klinik, Hofgarten 10, 34454 Bad Arolsen, Germany; 6 Research Unit for Telepsychiatry and e-Mental Health, Department of Clinical Research, University of Southern Denmark, J.B. Winsløws Vej 19, 5000 Odense, Denmark; 7 Department of Affective Disorders, Aarhus University Hospital - Psychiatry, Palle Juul Jensens Boulevard 175, 8200 Aarhus, Denmark; 8 Mental Health Unit, Barbastro Healthcare Sector, Aragón Healthcare Service –SALUD, Carretera N-240, 22300 Barbastro, Aragón Spain; 9 Centre for Innovative Medical Technology, Odense University Hospital, Sdr Boulevard 29, 5000 Odense C, Denmark; 10 Danish Centre for Health Economics, DaCHE, Department of Public Health, University of Southern Denmark, J.B. Winsløws Vej 9B, 5000 Odense C, Denmark; 11 The Public Health Service of Galicia -SERGAS, Service of Mental Health and Drug Addiction, Edificio Administrativo de San Lázaro, 15703 Santiago de Compostela, Spain; 12 Department of Research and Innovation, Badalona Serveis Assistencials, Plaça de Pau Casals 1, 08911 Badalona, Spain; 13 Department Medical Informatics, Middle East Technical University, Informatics Institute, Üniversiteler Mahallesi, Dumlupınar Bulvarı 1, 60800 Ankara, Turkey; 14 Ruhuna İyi Bak, Online Counseling Centre, Çağlayan Mahallesi Bahtiyar Sokak 50/1, 34403 Istanbul, Turkey; 15 ULSS9 Treviso, Via Scarpa, 9, 31100 Treviso, Italy; 16 Mental Health Department, ASLTO3, Local Health Authority Torino 3, Via Martiri XXX Aprile 30, 10093 Collegno, Italy; 17 Institute of health service research - Kronikgunem, Torre del Bilbao Exhibition Centre, Azkue Kalea, 1, 48902 Barakaldo, Basque Country Spain

**Keywords:** Internet interventions, Depression, System usability scale, Psychometric evaluation, Confirmatory factor analysis

## Abstract

**Background:**

The System Usability Scale (SUS) is used to measure usability of internet-based Cognitive Behavioural Therapy (iCBT). However, whether the SUS is a valid instrument to measure usability in this context is unclear. The aim of this study is to assess the factor structure of the SUS, measuring usability of iCBT for depression in a sample of professionals. In addition, the psychometric properties (reliability, convergent validity) of the SUS were tested.

**Methods:**

A sample of 242 professionals using iCBT for depression from 6 European countries completed the SUS. Confirmatory Factor Analysis (CFA) was conducted to test whether a one-factor, two-factor, tone-model or bi-direct model would fit the data best. Reliability was assessed using complementary statistical indices (e.g. omega). To assess convergent validity, the SUS total score was correlated with an adapted Client Satisfaction Questionnaire (CSQ-3).

**Results:**

CFA supported the one-factor, two-factor and tone-model, but the bi-factor model fitted the data best (Comparative Fit Index = 0.992, Tucker Lewis Index = 0.985, Root Mean Square Error of Approximation = 0.055, Standardized Root Mean Square Residual = 0.042 (respectively χ^2^_diff_ (9) = 69.82, p < 0.001; χ^2^_diff_ (8) _=_ 33.04, p < 0.001). Reliability of the SUS was good (ω = 0.91). The total SUS score correlated moderately with the CSQ-3 (CSQ1 r_s_ = .49, p < 0.001; CSQ2 r_s_ = .46, p < 0.001; CSQ3 r_s_ = .38, p < 0.001), indicating convergent validity.

**Conclusions:**

Although the SUS seems to have a multidimensional structure, the best model showed that the total sumscore of the SUS appears to be a valid and interpretable measure to assess the usability of internet-based interventions when used by professionals in mental healthcare.

## Background

### Implementation of iCBT

Mental healthcare in Western Europe is gradually being digitalized. Apart from administrative systems such as electronic patient records, professionals are being introduced to other digital services. These services can support or replace the delivery of regular treatment for mental disorders such as depression. Currently, one of the most studied treatments is internet-based cognitive behavioural therapy (iCBT) [1]. iCBT in a guided or blended format, has proven to be effective in the treatment of depression [2–4]. However, the translation of research findings and implementation to the complex field of routine mental healthcare practice is slow and challenging. As research showed that one of the barriers for implementation is the low usability of internet-based interventions, it is important to assess the usability of iCBT [5, 6]. In feasibility and evaluation studies on iCBT in mental healthcare, the System Usability Scale (SUS) has increasingly been applied to measure usability [7–9], however this instrument has not yet been validated in this emerging field.

### System usability

Although the meaning of usability is under debate (e.g. ) [10], usability can be seen as the perceived ‘ease of use’, ‘user-friendliness’ or ‘quality of use’ of a system, interface or product. In the international standard definition, usability is described as the extent to which a product can be used by specified users to achieve designated goals with effectiveness, efficiency, and satisfaction in a specified-context of use [11]. Satisfaction is related to system usability in the sense that satisfaction can contribute to the level of usability or where satisfaction is a consequence of usability [12]. The SUS is a popular instrument to measure the perceived usability of a wide range of products and systems. These include websites, apps, everyday products, software and hardware. Although the SUS has been presented as a ‘quick and dirty’ instrument [13], it is probably not that ‘dirty’ at all [14]. The SUS provides a single score for usability and is designed as a unidimensional (one factor) measurement [13]. In addition, it is without costs, technology agnostic, brief, reliable and valid [15]. Users are presented with ten statements that relate to various aspects of usability (i.e. need for support, complexity) on a 5-point Likert scale, ranging from strong disagreement (1) to strong agreement (5). The final score for the SUS ranges from 0 to 100, with higher scores indicating higher perceived usability.

### Psychometric properties and factor structure of the SUS

The psychometric properties of the SUS are sufficiently studied with reported reliabilities between 0.79 and 0.97 (e.g. )[16, 17], acceptable levels of convergent validity with other measures of perceived usability (e.g. ) [18] and sensitivity (e.g. ) [14]. Normative data is available based on scores from 11.855 individual SUS assessments from 166 (unpublished) industrial usability studies [19].

The original English SUS is formally translated into different languages: Arabic, Slovene, Polish, Italian, Persian, and Portuguese [15]. In addition, informal Dutch, French and Spanish translations are available [20]. Several studies added interpretation to the SUS scores: Bangor, Kortum and Miller [21] added an 7-point rating scale to the SUS to provide a SUS score with grades ranging from A to F. A score of 70 is for example given a ‘C’ which is considered ‘good’. Sauro and Lewis [22] published a curved grading scale with a score of 68 as the centre of the scale, that can be interpreted as a cut-off for above and below average usability scores.

As for the factor structure, Lewis and Sauro [14] proposed that there might be two factors in the SUS: Usability and Learnability. Since then, studies replicated inconsistent findings pointing towards this two-factor model (e.g. ) [23] and the one-factor model as well [24, 25]. More recent research showed that two-factor structure possibly depends on the amount of experience that users have with a given product [18]. The SUS acted as a one-factor scale with less product experience, but showed a two-factor structure when more time was spent with the product, in this case an e-learning platform. In 2017, Lewis and Sauro revisited the factor structure of the SUS and tried to replicate the two-factor structure [26]. However, they found a different two-factor structure produced by the positive and negative tone of the items. As the tone structure is of little practical and theoretical interest, their conclusion was to treat SUS as a single factor structure. They suggested that the Usability/Learnability structure can appear in certain circumstances, but that such findings require replication.

### Aim

The SUS has proven itself to be a useful instrument in an industrial context. However, it remains unclear whether it is a valid instrument in measuring the usability of guided and blended CBT applications, as perceived by professionals in the context of implementation within routine mental healthcare. Therefore, the aim of this study is to assess the different factor structures of the SUS, measuring usability of iCBT. Four models will be tested: (1) a single factor model to test whether the items in the questionnaire can be summarized by one single factor score, (2) a two-factor model to test whether Usability and Learnability are two different factors, (3) a tone model to test the effect of positive and negative items and (4) a bi-factor model to confirm whether the single factor is measured by all items as well as the factors Usability and Learnability by the indicated subsets of items. In addition, the psychometric properties (reliability, convergent validity) of the SUS will be tested.

## Methods

### Recruitment

The professionals were recruited in the context of the large scale European implementation project MasterMind that aimed to provide a summative evaluation of the factors related to uptake of unguided, guided and blended iCBT in 14 regions in 10 countries [27, 28]. The project explored the role of different stakeholders that were involved in the implementation of the intervention (e.g. patients, professionals and representatives of mental healthcare organizations). For the purpose of this study, the data provided by the professionals was used (*n* = 242). They provided guided and blended iCBT to patients with depressive symptoms in the Netherlands (*n* = 51), Germany (*n* = 16), Denmark (*n* = 4), Spain (*n* = 135), Italy (*n* = 33) and Turkey (*n* = 3). Data from the other MasterMind countries (Scotland, Wales, Estonia and Norway) were not suitable for the purpose of this study, as these countries evaluated unguided iCBT and the professionals’ interaction with the interface of iCBT was very minimal.

### SUS questionnaire

Via online and paper-based surveys, professionals were asked to rate the usability of iCBT interventions using ten items of the SUS on a 5-point self-report scale, ranging from 1 (I strongly disagree) to 5 (I strongly agree) after 18 months of data collection within the MasterMind study. The professionals had different levels of iCBT experience when the SUS was administered. Five statements were positively formulated (items with odd numbers) and five statements negatively (items with even numbers). See Table 1 for the description and response categories of the SUS items, adapted to the MasterMind study and the use of iCBT. In the countries where no translation to the local language was available, the forward and backward method was followed to translate the SUS items (i.e. first the questionnaire was translated from English into the local language by two persons who reached consensus by discussion. Then the questionnaire was translated back to English and was compared with the original questionnaire).

**Table 1:** Description of the SUS items and response categories in the MasterMind study

SUS items		*I strongly disagree*	*I disagree*	*I don’t disagree nor agree*	*I agree*	*I strongly agree*
1.	I think that I would like to provide the iCBT intervention to my clients more frequently.	•	•	•	•	•
2.	I found the iCBT intervention unnecessarily complex.	•	•	•	•	•
3.	I find the iCBT intervention easy to use in treating my clients.	•	•	•	•	•
4.	I think that I would need the support of a technical person to be able to use and provide the iCBT intervention to my clients.	•	•	•	•	•
5.	I found the various functions in the iCBT intervention were well integrated.	•	•	•	•	•
6.	I thought there was too much inconsistency in the iCBT intervention.	•	•	•	•	•
7.	I can imagine that most healthcare professionals would learn to use and provide the iCBT intervention very quickly.	•	•	•	•	•
8.	I found the iCBT intervention very cumbersome to use.	•	•	•	•	•
9.	I felt very confident using and providing the iCBT intervention to my clients.	•	•	•	•	•
10.	I needed to learn a lot of things before I could get going with using and providing the iCBT intervention to my clients.	•	•	•	•	•

To calculate the overall SUS score, the following formula was applied [13]: The item score on the positive statements was subtracted by 1 (x-1) and the item score on the negative statements was calculated by subtracting the score from 5 (5-x). The sum of these item scores was then multiplied by 2.5 to provide an overall SUS score between 0 (extremely poor usability) and 100 (excellent usability). The subscale Learnability consists of the items 4 and 10 and the subscale Usability of the remaining items.

### CSQ-3 questionnaire

To assess convergent validity, three questions that were adapted from the Client Satisfaction Questionnaire (CSQ-3) [29] were used. See Table 2 for a description of the items and response categories. Same as with the SUS items, in the countries where no translation to the local language was available, the back-translation method was followed to translate the CSQ items [30]. The three items of the CSQ are the main items for measuring overall satisfaction of health and human services and is frequently used as one measure among a battery of other instruments. The CSQ shows good reliability and validity and is used across a range of services, from inpatient to forensic services, without a specific setting of care [31] and internet-based interventions [32]. Each item on the CSQ-3 is scored from 1 (low satisfaction) to 4 (high satisfaction).

**Table 2:** Description of the CSQ-3 items and response categories in the MasterMind study

CSQ-3 items			
1. To what extend has the iCBT intervention met your needs in treating depressed patients?			
•	•	•	•
*None of my needs have been met*	*Only a few of my needs have been met*	*Most of my needs have been met*	*Almost all of my needs have been met*
2. In an overall general sense, how satisfied are you with the iCBT treatment you have provided?			
•	•	•	•
*Quite dissatisfied*	*Indifferently or mildly dissatisfied*	*Mostly satisfied*	*Very satisfied*
3. If you were to provide treatment again, would you use the iCBT intervention again?			
•	•	•	•
*No, definitely not*	*No, I don’t think so*	*Yes, I think so*	*Yes, definitely*

### Statistical analyses

Data of six European countries were pooled for analytic purposes. Statistical analysis was carried out using RStudio (v1.2.1335; RStudio Team, 2015) using the packages *lavaan* [33], *psych* [34] and *subscore* [35].

To assess the factor structure, a Confirmatory Factor Analysis (CFA) was conducted. Four models were evaluated: the one-factor model, the two-factor (Usability/Learnability) model, the tone model (positive/negative) and a bi-factor model. Due to the application of the five-point Likert scale, the responses to the SUS-items were considered ordinal data. Hence, the Weighted Least Squares Mean and Variance adjusted (WLSMV) estimator was used as a method of parameter estimation as this is recommended for the analysis of ordinal data [36]. Overall model fit was assessed using a set of goodness-of-fit indices and criterion values, as suggested by Brown [36] as these indices provide an overall satisfactory performance in evaluating models: Chi-square (χ^2^), Comparative Fit Index (CFI, close to 0.95 or greater), Tucker Lewis Index (TLI, close to 0.95 or greater), Root Mean Square Error of Approximation (RMSEA, close to 0.06 or below) and Standardized Root Mean Square Residual (SRMR, close to 0.08 or below). These fit indices were considered in combination, as a good fit meets all the chosen criteria [37]. A scaled chi-square difference test was applied to compare the fit of the two models [36].

To further investigate the SUS structure and to assess the reliability of the SUS, more advanced statistics were calculated in order to evaluate the found factor structures in the context of finding the best solutions. The omega coefficient was calculated together with other indices: the percentage of uncontaminated correlations (PUC), the explained common variance (ECV), and omega hierarchical [38]. Omega is a reliability estimate that does not depend on the assumption of tau equivalence unlike its classic counterpart Cronbach’s alpha [39]. There is no cut-off point for omega to evaluate acceptable reliability, a minimum of .50 and values closer to .75 are recommended for satisfactory and good reliability [40]. PUC is the percentage of covariance terms which only reflect variance from the general dimension. ECV is the proportion of all common variance explained by the general factor. Along with ECV, PUC influences the parameter bias of the unidimensional solution. When PUC is greater than .70 and ECV greater than .70 relative bias will be slight and the common variance can be regarded as essentially unidimensional [39]. When a PUC value is lower than .80, the general ECV value greater than .60 and omega hierarchical (of the general factor) is greater than .70 it is suggested that the presence of multidimensionality is not severe enough to disqualify the interpretation of the instrument as primarily unidimensional [40]. To indicate more precisely whether the subscales has added value over and above the total SUS score Haberman introduces a methodology to qualify this added value. This is done by computing the proportional reduction in mean squared error (PRMSE) based on the total score (PRMSE_total_) and comparing that value to the proportional reduction in mean squared error based on subscale scores (PRMSE_subscale_) [41]. If the ratio of these values, subscore over total score, exceed one, the subscore does not have added value and it is not recommended to use the subscore in statistical models.

To assess convergent validity, Spearman’s rank-order correlations were calculated between all three items of the CSQ-3 and the total SUS score. A common criterion for the absolute magnitude of correlations that supports the hypothesis of convergent validity is a minimum of 0.30 [42].

### Power analyses

Estimating the required sample size for CFA is complex because various aspects (e.g. study design, missing data level, scaling, estimator type, model complexity) need to be taken into account and can vary widely from data set to data set [36]. The existing literature provides limited and sometimes conflicting rule of thumb for the required sample size for CFA [43]. A sample size of > 200 seems sufficient for robust parameter estimations with ordinal data according to Bandalos and Forero and colleagues [44, 45]. In which sample sizes between 200 and 500 are recommended.

## Results

### Sample

The respondents (*n* = 242) completed the questionnaire between August and December 2016. Table 3 provides an overview of the respondent characteristics. Most respondents were female (71.5%), 39.3% of the respondents were GP’s, 34.7% were psychologists or psychiatrists and 24.8% had a different professional background (e.g. specialized health nurses, health workers). About half of the respondents had more than 5 years of professional experience in mental healthcare (54.5%). The majority of respondents (57%) had provided iCBT at least five times to treat depressed patients. Still, a relatively large group (36.4%) had little experience with iCBT (i.e. providing iCBT less than 5 times to patients).

**Table 3:** Sample characteristics of respondents

Variable	Label	Pooled, n (%)
N	Cases	242 (100)
Gender	Female	173 (71.5)
	Missing	2 (0.8)
Profession	GP	95 (39.3)
	Psychologist	76 (31.4)
	Psychiatrist	8 (3.3)
	Other	60 (24.8)
	Missing	3 (1.2)
Field experience	0–2 years	69 (28.5)
	3–4 years	36 (14.9)
	5–9 years	39 (16.1)
	> 10 years	93 (38.4)
	Missing	5 (2.1)
iCBT experience	1–4 times	88 (36.4)
	5–9 times	48 (19.8)
	10–14 times	23 (9.5)
	15–19 times	24 (9.9)
	> 20 times	43 (17.8)
	Missing	16 (6.6)

### SUS and CSQ scores

**Fig. 1:**
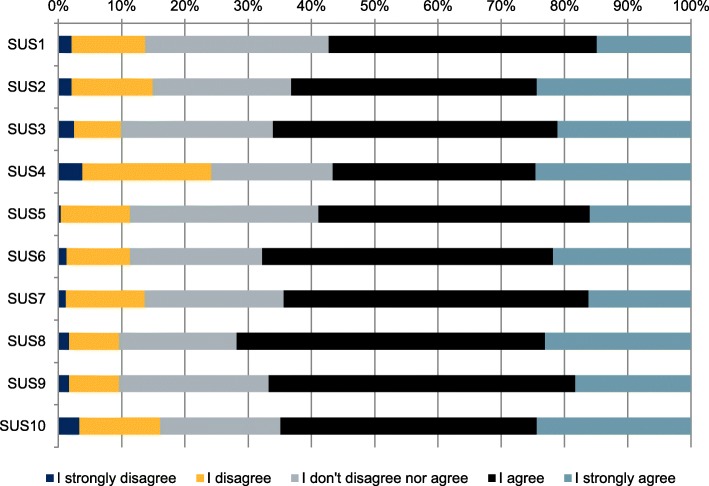
Percent distribution of item responses on the (inversed) SUS items

The pooled mean total score of the SUS was 67.9 (SD 16.3; range 20–100), indicating a (just) below average score. See Tables 4 and 5 for mean scores of the SUS and the CSQ-3. Figure 1 gives a visual overview of the distribution of the item responses on the SUS. See Additional file 1 for a percentile rank of SUS items scores, a covariance matrix of SUS item scores and the distribution of the frequencies of the total SUS scores.

**Table 4:** Mean, standard deviation and range of the (recoded) SUS scores

	Pooled, mean (SD; range)		
N	242		
SUS1	3.56 (0.95;1–5)	SUS2	3,71 (1.04;1–5)
SUS3	3.75 (0.96;1–5)	SUS4	3,53 (1.18;1–5)
SUS5	3.63 (0.89;1–5)	SUS6	3,77 (0.95;1–5)
SUS7	3.66 (0.94;1–5)	SUS8	3,84 (0.93;1–5)
SUS9	3.74 (0.91;1–5)	SUS10	3,70 (1.08;1–5)
SUS total	67.85 (16.28;20–100)

**Table 5:** Mean, standard deviation and range of the CSQ-3 scores

	Pooled, mean (SD; range)
N	241
CSQ1	2.91 (0.78;1–4)
CSQ2	3.10 (0.66;1–4)
CSQ3	3.28 (0.68;1–4)

### Confirmatory factor analysis

**Fig. 2:**
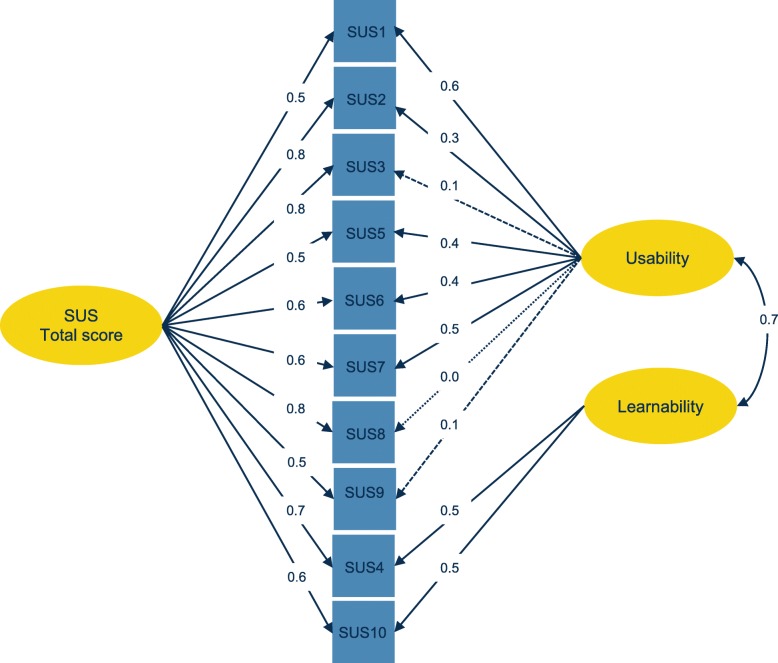
Factor structure of the bi-factor model of the SUS

Table 6 shows the results for the four models. The overall goodness-of-fit indices suggested that the one-factor, the two-factor and tone models have an acceptable fit (i.e. CFI ≥ 0.95, TLI ≥ 0.95, SRMR ≤0.08). However, further inspection revealed a better fit for the indices of the bi-factor. The scaled chi-square test confirmed that the difference between the bi-factor model and the other models was statistically significant (one-factor *χ*^*2*^_*diff*_ = 69.82, *df* = 9, *p* < 0.001; two-factor *χ*^*2*^_*diff*_ = 33.04, *df* = 8, *p* < 0.001; tone model *χ*^*2*^_*diff*_ = 59.58, *df* = 8, *p* < 0.001). Fig. 2 shows a visualization of the bi-factor model, with a positive correlation (*r*_*s*_ = 0.70) between the factors Usability and Learnability. See Additional file 1 for an overview of the other models in diagrams and factor loadings.

**Table 6:** Results of the confirmatory factor analysis

Model	N_par_	Chi square	DF	CFI	TLI	RMSEA (CI)	SRMR
One-factor	50	124.84	35	0.960	0.949	0.103 (0.084–0.123)	0.079
Two-factor	51	82.19	34	0.979	0.972	0.077 (0.056–0.098)	0.066
Tone-model	51	109.88	34	0.966	0.955	0.096 (0.076–0.117)	0.075
Bi-factor	60	44.96	26	0.992	0.985	0.055 (0.026–0.081)	0.042

*Npar* number of parameters estimated in the CFA, *DF* degrees of freedom, *CFI* Comparative Fit Index, *TLI* Tucker Lewis Index, *RMSEA* Root Mean Square Error of Approximation, *CI* 90% confidence interval, *SRMR* Standardized Root Mean Square Residual

### Reliability

The reliability analysis showed that the 10 items on the SUS had acceptable reliability, ω = 0.91. The PUC was 0.36, the ECV was 0.75 and omega hierarchical was 0.78, meaning that the SUS is not entirely convincingly unidimensional and at the same time the presence of the two subscales (Usability/Learnability) not serious enough is to disqualify undimensionality. This was made clear by the PRMSE results of the total (PRMSE_total_ = 0.93 and subscale scores (PRMSE_usability_ = 0.70, PRMSE _learnability_ = 0.55): both PRMSE ratio values exceed 1 confirming that the subscales do not have added value over the total score.

### Convergent validity

The total score of the SUS correlated moderately with the three items on the CSQ-3, indicating convergent validity between the two measures (See Table 7).

**Table 7:** Convergent validity of SUS and CSQ-3

Item	Spearman correlation with SUS total score (CI)
CSQ1. Have the needs been met?	0.49 (0.39 to 0.58)^a^
CSQ2. Overall satisfaction?	0.46 (0.35 to 0.55)^a^
CSQ3. Provide treatment again?	0.38 (0.26 to 0.48)^a^

*CI* 95% confidence interval, ^a^Correlation is significant at 0.001

## Discussion

Usability of internet-based interventions are an important factor in successful implementation and patient engagement [6]. Findings of our study demonstrate that the System Usability Scale (SUS) is a valid measure to assess the usability of iCBT in mental healthcare.

The CFA provided support for the bi-factor model; this model fitted the data better than the one-factor model, the two-factor model or the tone model. Although this would mean that the SUS gives a score for overall usability, as well as scores for the subscales Usability and Learnability, further analysis showed that the subscales contain no information that is not already contained in the total score.

There may be several reasons why previous studies found mixed findings of the subscales Usability and Learnability. An explanation by Borsci [18] is that it depends on the level of ‘product’ experience or exposure. In our sample, the amount of iCBT experience among the professionals varied considerably. However, a large variety of product experience was also reported in studies that found a one-factor model (e.g. ) [24, 25]. Another explanation may be related to the complexity of the product; it can be assumed that the Learnability factor has more weight in a context that requires more learning (e.g. an e-learning or intervention platform) than a more straightforward context (e.g. microwave or mobile app). In the case of iCBT, professionals have to adapt to a new system and learn how to integrate this into their work routine. However, as Lewis also reported [26], the contexts in which Usability and Learnability (dis) appear need further investigation. Furthermore, the SUS research field could consider assessing complementary statistical indices with applying bi-factor models and its tendency to ‘overfit’, to make more informed decisions [46]. The correlations between the SUS and CSQ-scores indicated convergent validity, comparable to other studies that also found considerable evidence for the overlap with other related questionnaires such as the Usability Metric for User Experience (UMUX) [18, 47]. Moreover, the SUS in this study had a good reliability. This is in line with previous research in other contexts as well (e.g. ) [26].

There are several limitations in this study that need to be discussed. First of all, the sample had an uneven distribution of professionals per country resulting in an under and over representation of the countries included within the study. This also limited the analysis of the factor structure in taking into account the different countries and the possible biased standard error. Plus, the technical formats of the iCBT applications as well as the content of the iCBT interventions differed between countries. Hence, the representativeness of the sample might be affected by this. Secondly, as most translations of the original SUS items were informal, possible different interpretations by the professionals may have occurred. On the other hand, this risk was minimized by using a back-translation method [30]. It is possible that the factor structure of the SUS is distorted by the mixed tone of negatively and positively worded items. The mixed tone was originally used to control for acquiescence bias; the hypothesized tendency of respondents to agree with statements with a mix of positive and negative tone [19]. However, several studies encountered an unintentional SUS factor structure caused by the mixed tone (e.g. ) [24]. Moreover, there is evidence that the mixed tone caused respondents to make mistakes and researchers to miscode the questionnaire [48]. In 2011, Sauro and Lewis tested a positive version of the SUS and found no significant difference between the mean overall SUS scores of the positive and mixed versions [48]. In addition, they found no evidence for strong acquiescence bias or extreme response bias. To avoid problems caused by tone, future researchers could consider alternative formats of the SUS (e.g. a positive version, item specific response options, expanded scale format) [49, 50].

## Conclusions

This study demonstrated that the SUS had good psychometric properties, even in a heterogeneous sample of professionals in mental healthcare. Different factor structures were studied with reasonable outcomes. However the bi-factor model showed the best results in this sample indicating that researchers interested in the usability of internet-based interventions in mental healthcare can use the proposed scoring of the SUS and in particular the calculated sumscore.

## Supplementary information

**Additional file 1 **Table 1 Percentile rank of SUS items scores. Table 2 Covariance matrix of SUS item scores, *n* = 242. Figure 1 Distribution of frequencies of total SUS scores. Figure 2 Factor structure of the one-factor model, two-factor, and tone-model of the SUS.

## Data Availability

The data that support the findings of this study are available from [the MasterMind consortium] but restrictions apply to the availability of these data, which were used under license for the current study, and so are not publicly available. Anonymous data are however available upon reasonable request from the MasterMind publication committee. Please address your requests to Ebert, D.D. d.d.ebert@vu.nl.

## Abbreviations

CFA: Confirmatory Factor Analysis
CFI: Comparative Fit Index
CI: Confidence interval
CSQ: Client Satisfaction Scale
DF: Degrees of freedom
ECV: Explained common variance
iCBT: Internet-based Cognitive Behavioural Therapy
M: Mean, average
PRMSE: Proportional reduction in mean squared error
PUC: Percentage of uncontaminated correlations
RMSEA: Root Mean Square Error of Approximation
SD: Standard Deviation
SRMR: Standardized Root Mean square Residual
SUS: System Usability Scale
TLI: Tucker Lewis Index
WLSMV: Weighted Least Square Means and Variances
