# Supplementary material for: Dimensionality of the system usability scale among professionals using internet-based interventions for depression: a confirmatory factor analysis
Source: BMC Psychiatry. 2020 May 12;20:218. doi: 10.1186/s12888-020-02627-8 (PMC7216472; doi:10.1186/s12888-020-02627-8)
Supplement: Supplementary file 1 — Additional file 1 Table 1 Percentile rank of SUS items scores. Table 2 Covariance matrix of SUS item scores, n = 242. Figure 1 Distribution of frequencies of total SUS scores. Figure 2 Factor structure of the one-factor model, two-factor, and tone-model of the SUS. [file 12888_2020_2627_MOESM1_ESM.docx]

## **Additional file 1**

**Table 1** Percentile rank of SUS items scores.

| Percentile rank | SUS total score |
| --- | --- |
| 5^th^ | 37.5 |
| 10^th^ | 45.0 |
| 25^th^ | 57.5 |
| 50^th^ | 70.0 |
| 75^th^ | 78.8 |
| 90^th^ | 87.5 |
| 99^th^ | 95.0 |

**Table 2** Covariance matrix of SUS item scores, n=242.

| Item | SUS1 | SUS2 | SUS3 | SUS4 | SUS5 | SUS6 | SUS7 | SUS8 | SUS9 | SUS10 |
| --- | --- | --- | --- | --- | --- | --- | --- | --- | --- | --- |
| SUS1 | 0.905 |  |  |  |  |  |  |  |  |  |
| SUS2 | 0.483 | 1.080 |  |  |  |  |  |  |  |  |
| SUS3 | 0.373 | 0.577 | 0.911 |  |  |  |  |  |  |  |
| SUS4 | 0.389 | 0.633 | 0.525 | 1.380 |  |  |  |  |  |  |
| SUS5 | 0.393 | 0.418 | 0.341 | 0.367 | 0.799 |  |  |  |  |  |
| SUS6 | 0.405 | 0.583 | 0.368 | 0.388 | 0.414 | 0.892 |  |  |  |  |
| SUS7 | 0.479 | 0.525 | 0.401 | 0.415 | 0.376 | 0.424 | 0.877 |  |  |  |
| SUS8 | 0.289 | 0.529 | 0.499 | 0.464 | 0.304 | 0.354 | 0.307 | 0.858 |  |  |
| SUS9 | 0.300 | 0.253 | 0.305 | 0.233 | 0.157 | 0.199 | 0.312 | 0.349 | 0.819 |  |
| SUS10 | 0.146 | 0.496 | 0.434 | 0.713 | 0.177 | 0.236 | 0.209 | 0.445 | 0.249 | 1.158 |

**Fig. 1** Distribution of frequencies of total SUS scores.

**Fig. 2** Factor structure of the one-factor model, two-factor, and tone-model of the SUS.
